# Nutrition as an etiological factor causing diseases in endangered huemul deer

**DOI:** 10.1186/s13104-020-05122-1

**Published:** 2020-06-08

**Authors:** Werner T. Flueck

**Affiliations:** 1 Swiss Tropical and Public Health Institute, University Basel, Socinstrasse 57, 4051 Basel, Switzerland; 2 National Council of Scientific and Technological Research (CONICET), Buenos Aires, Argentina; 3 Argentine National Park Administration, Rolando 699, 8400 Bariloche, Argentina

**Keywords:** Huemul, Hippocamelus bisulcus, Osteopathology, Clinical evaluation, Parapoxvirus, Selenium, Copper, Manganese

## Abstract

**Objectives:**

Distinct diseases prevent endangered huemul deer (*Hippocamelus bisulcus*) recovery. Fundamental etiological factors include nutriments, a mayor component of habitat quality. Undernutrition affects growth, skeletal development, osteopathology, reproduction and immunocompetence: this paper amplifies data corroborating micro-nutrient deficiencies among huemul.

**Results:**

In Argentina, 57% huemul cadavers exhibited osteopathology, with new cases reported here. Recently, 86% live huemul had osteopathology: cranial lesions involved antemortem tooth loss, reducing feeding efficiency and body condition, with starvation deaths. This population had tissues well deficient compared to other cervids, averaging 0.28 ppm selenium, 4.98 ppm copper, whereas for manganese 55% were deficient (2.52 ppm) and 45% adequate (42.79 ppm). Recently, lesions in one Chilean huemul were interpreted to stem from parapoxvirus. That population also has cases with cranial osteopathologies, high disease susceptibility (parapoxvirus, parasitism, foot lesions), crippled antlers, and low density, indicative of marginal habitat and primary etiological factors like undernutrition and immunosuppression. The reported atypical symptoms attributed to parapoxvirus may relate to probable diagnostic limitations, but does support presence of nutritional deficiencies. Patagonia has selenium deficient plants and livestock, including severe muscular dystrophy, and soil levels in extant huemul areas considered very deficient. Moreover, 73% of Chilean huemul were selenium deficient and 64% severely deficient with concomitant cranial osteopathology.

## Introduction

Patagonian huemul deer (*Hippocamelus bisulcus*) is highly endangered: 350–500 in Argentina, some 1000 in Chile, and with a diminishing trend [1, 2]. Huemul have distinct diseases, interpreted to contribute to a failing recovery: e.g. osteopathology [3–10], and recently, foot lesions from Bernardo O’Higgins National Park, Chile (BONP) [11]. Describing the first-ever parapoxvirus (PPV) infection in a single huemul, resulting foot lesions were concluded to pose considerable conservation risks [11]. Although reliable findings essentially require replicated research [12], this is precluded in rare wildlife, frequently not achieving but single focal studies.

Besides limiting factors commonly outlined for huemul (small subpopulations, multi-host pathogens, reservoir hosts, pathogen characteristics, factors affecting transmission, climate change) [11], another fundamental etiological factor is nutrition, a mayor component of habitat quality [9]. Micro-nutrient deficiencies are well recognized, affecting body growth, skeletal development, reproduction and immunocompetence [13]. Distinguished micro-nutrients contributing to immune balance include zinc (Zn), copper (Cu), manganese (Mn), and selenium (Se) [14–16]. Copper deficiency increases susceptibility to parasitic, bacterial, and viral infection, and may cause bone and hoof problems as found in cervids [15, 17, 18]. Se deficiency significantly reduces host defense, but also impairs osteometabolism [7, 19], including hooves [18]. Additionally, Se influences all immunological components, their responses to infections and cancer [8, 20, 21], and deficiency associates with increased incidence, severity (virulence) and/or progression of viral infections such as influenza, HIV and Coxsackie virus [21–23]. New data reported here corroborates the high prevalence of osteopathology, and key role of undernutrition among huemul in impairing immunocompetence and osteometabolism [3–9].

## Main text

### Methods

Analyses are based on field and laboratory results and tomography. Huemul in the Protected Park Shoonem (PPS: Chubut-Argentina) were marked after sedation with medetomidine-ketamin, and reversed by atipamezole. Necropsies were performed using standard techniques [24, 25]. For elemental analysis, samples were washed and dried at < 60 ℃. After digestion, samples were analyzed with Agilent 8800 ICP Triple Quad [26]. Tomography was obtained using GE-Discovery 710 PET/CT and MRI GE-SIGNA PET/MR 3 Tesla.

## Results

### Osteopathological lesions

**Fig. 1:**
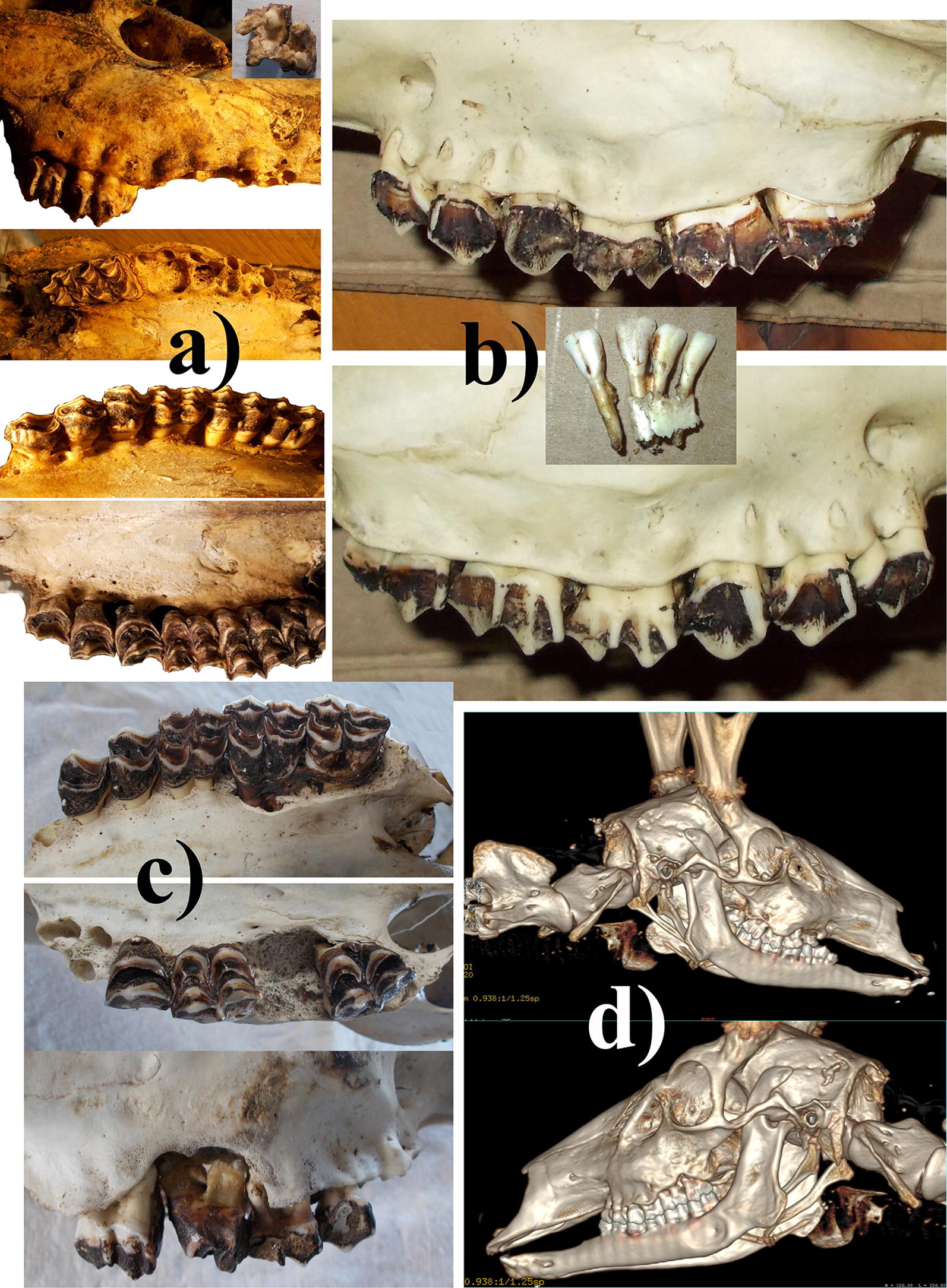
**a** Male that died in mid summer: retreated lingual margins of the palate; palatal perforations from thinning; partially exposed dental roots (arrow, bottom). Vanished alveoli on the right side with M2 and M3 very loose, whereas M1 and premolars directly fall out (top). Moreover, roots of the M1 and P4 are covered with solid exostosis (insert). **b** Male that died at the end of autumn: retreated maxillary line on the buccal side, resulting in partially exposed dental roots; extremely thin and even absent maxillary buccal walls, with roots partially exposed in 50% of maxillary teeth; mandibular alveoli for incisive teeth are thin walled and so short that the upper 40% of the roots are exposed. **c** Male that died in early summer: retreat of both lingual margins of the palate, resulting in exposed dental roots, and palatal perforations from thinning; alveoli and maxillary wall so reduced on right side has that molars are completely loose; roots covered with solid exostosis. **d** Male that died at beginning of spring: had lost 7 of 8 incisors in vivo; receded bone around maxillary roots which are exposed, loss of all right maxillary premolars; mandibula with depression containing pus and perforations; left mandible with severe exostosis on buccal side and bone resorption including perforations on buccal and lingual side

A male skeleton with hard antlers was found on the lake shore (PPS). Based on attached tissues (skin, fur, tendons, muscle), he died during February (summer). Several bones had scavenger and/or predator marks. Initial skeleton inspection, however, revealed severe cranial pathology. Both lingual margins of the palate have retrieved substantially, resulting in partially exposed dental roots, and palatal perforations from thinning (Fig. 1a). Alveoli on the right side have vanished such that M2 and M3 are very loose, whereas M1 and premolars directly fall out (Fig. 1a). Moreover, roots of M1 and P4 are covered with solid exostosis (Fig. 1a).

A near-complete male carcass with hard antlers (3.5 years old) was found on a beach (PPS). Based on skin, muscles, absence of odor and bloating, he died in late autumn, without signs of predation and limited bird scavenging. Severe cranial pathology include a retracted maxillary line on the buccal side, resulting in partially exposed dental roots (Fig. 1b). Furthermore, maxillary buccal walls are extremely thin and even absent, such that roots of 50% of teeth are partially exposed (Fig. 1b). Moreover, mandibular alveoli for incisive teeth are thin-walled and so short that the upper 40% of roots are exposed (Fig. 1b). Lastly, antlers are asymmetrical which frequently indicates osteological problems.

A male radio-collared in winter had clinical problems with walking. He survived 2.5 years when found by a creek where he died in early summer (4–5 years old). Signs of depredation were absent, yet scavengers had separated body parts, but all remains were within 15 m. Initial skeleton inspection revealed severe cranial pathology. Both lingual margins of the palate have retrieved substantially, resulting in partially exposed dental roots, and palatal perforations from thinning (Fig. 1c). The right side also has alveoli and maxillary wall so reduced that molars are completely loose, and moreover, the roots are covered with solid exostosis (Fig. 1c). Lastly, marrow fat depots were absolutely lacking, indicating the emaciated state and starvation as likely mortality cause.

A male radio-collared in spring had clinical problems of premature tooth loss (7 of 8 incisive teeth) and swellings due to bilaterally severely affected mandibles (3–4 years old). His fresh carcass laying on a beach the following spring, lacked signs of depredation. However, advanced muscle catabolism, lack of fat depots with only 11.8% left in bone marrow, and emaciated state indicated starvation as mortality cause. Besides loss of incisors, skull analysis revealed also premature loss of mandibular molars and maxillary premolars, exposed tooth roots, and severe bone remodeling (Fig. 1d).

### Micro-nutrient status

Hair from huemul in PPS (n = 11) revealed micro-nutrient deficiencies, with averages of 0.282 ppm for Se (SE 0.056), and 4.984 ppm for Cu (SE 0.54). Regarding Mn, 6 animals averaged 2.510 ppm (SE 0.511), whereas the other 5 cases averaged 42.788 ppm (SE 4.751) (Additional file 1).

## Discussion

### Nutrition affecting bone metabolism and immunocompetence

#### Crucial osteopathological lesions

**Fig. 2:**
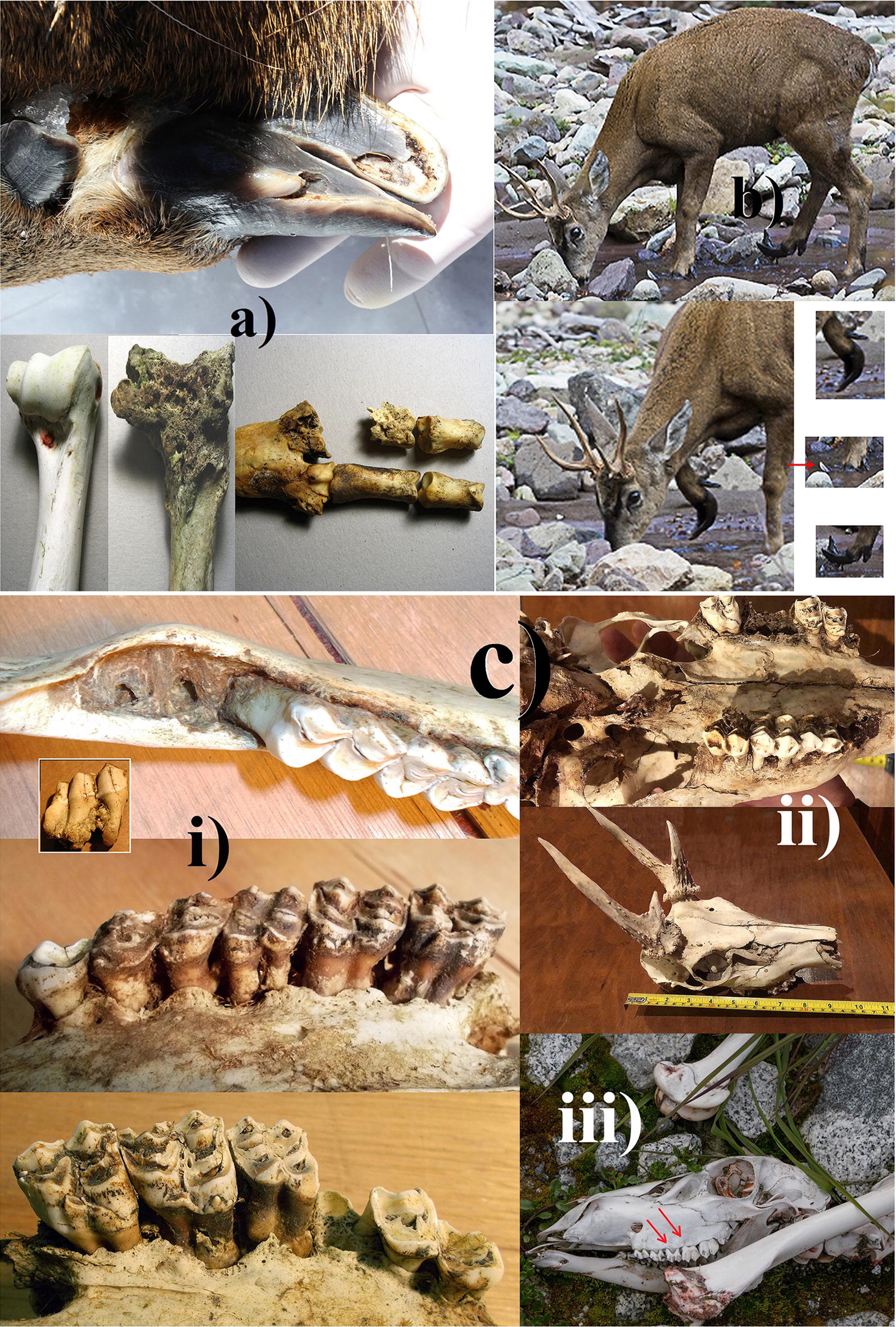
Foot lesions in huemul: **a** Protected Park Shoonem, Argentina: lesions in vivo include extensive tears (3 cm) on the abaxial wall of the hoof, reaching under the frontal third of the callus pad; and with the subunguinus partially detached and missing (above). Remains of dead huemul revealed articular lesions, like in feet and humerus (below). **b** Chilean populations: a male observed very weak and with foot lesions, died shortly after. At least three feet exhibited hoof lesions (photos taken by C. Panichine Faundes). **c** Huemul heads with lesions from Bernardo O’Higgins National Park (Chile): (i) head of female observed in 1993: with perforations on buccal and palatal sides of maxillary bone, exposing root apices of premolar and molar teeth; exposed roots of maxillary teeth from generalized erosion and resorption of bone resulting in enlarged dental alveoli; crystalline deposits on tooth roots (insert); resorption as well as osteomyelitic thickening of mandibular body. (ii) Male head from 2017: perforations on buccal and palatal sides of maxillary bone, exposing root apices of premolar and molar teeth; exposed roots of maxillary teeth from generalized erosion and resorption of bone resulting in enlarged dental alveoli (above). Antler development (below) qualifies as subnormal, indicating severe nutritional limitations for the annual cycle of antler regrowth. (iii) Juvenile female from 2019: resorption on buccal side of maxillary bone, exposing roots of premolar and molar teeth (Ultima Patagonia 2019, www.centre-terre.fr Accessed 10 July 2019)

Argentina: huemul with drastic cranial lesions frequently also exhibited appendicular lesions [3–6, 10]. Such lesions occurred in several subpopulations: 57% of adults with osteopathology, with legs affected in 45% [3, 4, 6]. Only recently confirmed in PPS, 86% of live huemul had bone lesions, and cranial lesions involved antemortem tooth loss, reducing feeding efficiency and body condition (Fig. 1d) [4]. Live cases also evidenced lameness and affected hooves (Fig. 2a), promoting secondary infections, but certainly inflammation and necrosis. The four new cases from PPS described here corroborate the high prevalence of osteopathology among huemul, at young ages, and resulting in starvation deaths.

Chile: a male with abnormal hooves was necropsied (Fig. 2b) [27]. For huemul with foot lesions and one case with suspected PPV infection (BONP), chronic osteopathology was discarded [11]: however, the absence of revisions nor preservation of appendicular bones and skulls of those diseased huemul prevents a comparison with osteopathological processes described elsewhere [3–6, 10, 28]. Yet similar cranial lesions also occur in Chile [5]. Besides foot lesions (2005–2010) in BONP [11], although not mentioned in [11], cranial lesions actually occurred in BONP: two cases reported by Texera [28]; a female analyzed with A. Frid (Fig. 2c); a young male (2017) with advanced lesions and underdeveloped antlers (Fig. 2d); and a juvenile (2019) with maxillary bone resorption (Fig. 2e). These cranial lesions express pathological processes of longer duration than described regarding foot lesions [11].

**Fig. 3:**
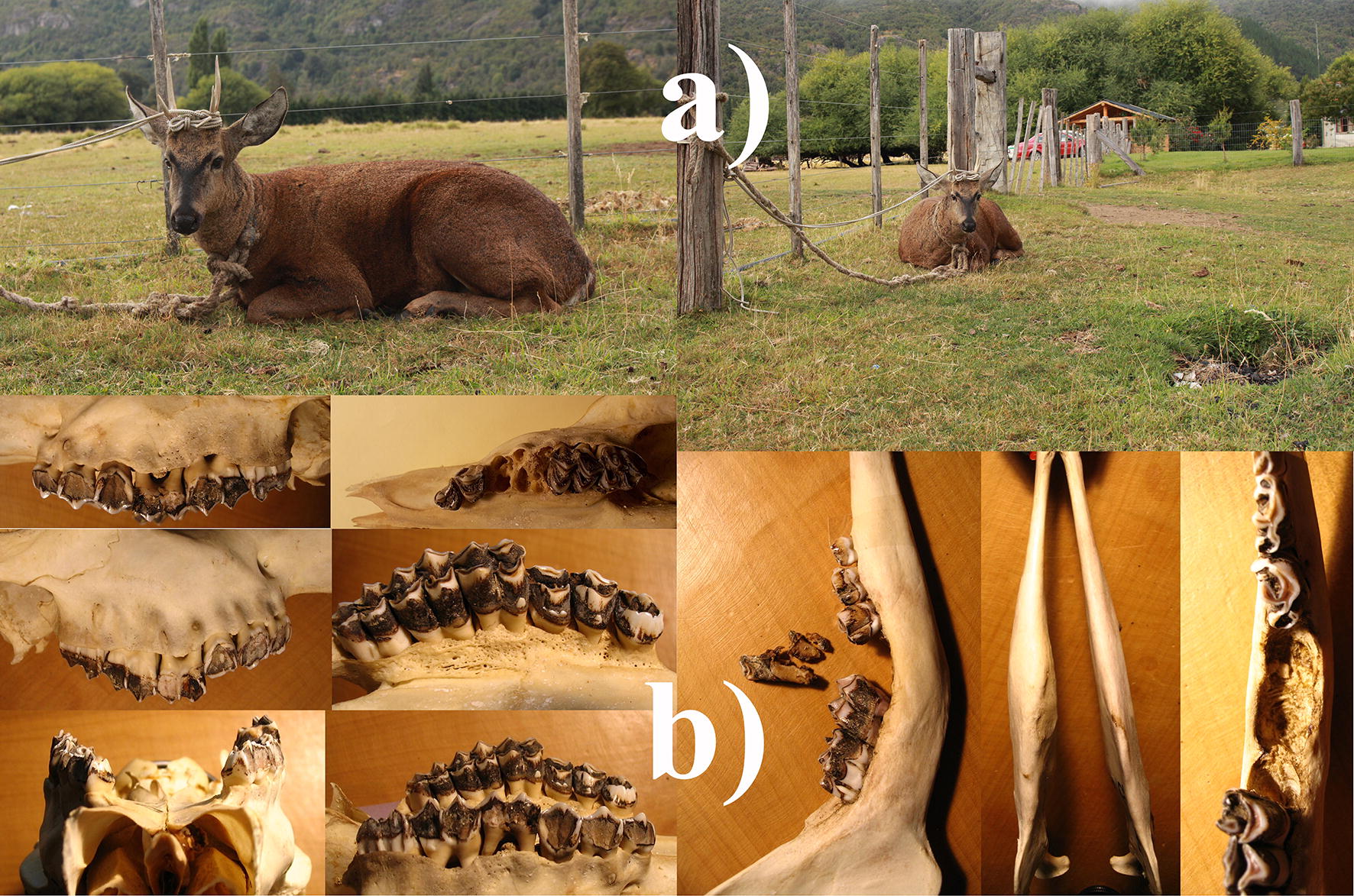
Misdiagnoses in live and dead huemul: **a** sick huemul apparently in healthy state; **b** initially reported without health problems, a subsequently necropsy showed severe lesions. Maxillary (left): recessed, porous and perforated bone on labial sides, necrotic alveoli and exposed roots such that M1, M2 and P4 fall out in the absence of soft tissue, the right maxillary molars protrude some 6 mm more than the left teeth row due to altered mandible. Mandible (right): reduced height and thickened body of the right body due to osteolysis with ventral border bent excessively during bone restructuring, absent alveoli, fractured M1 with pieces merely held by soft gum tissue, misaligned M2

Severe cranial lesions occur in subpopulations spread along 1000 km of the Andean mountains (Flueck and Frid unpublished,10,28). Lastly, misdiagnoses in live and dead huemul are not uncommon, also because sick huemul frequently pretend a healthy state (Fig. 3a). For instance, a male reported as healthy showed severe cranial lesions once necropsied (Fig. 3b).

#### Micro-nutrients relevant for bone metabolism and immunocompetence

In Argentina, the present results from huemul reveal for the first time that concentrations of several micro-nutrients are well below those considered deficient as determined for other cervids (hair): < 0.5 ppm Se, < 6.7 ppm Cu, and < 7 ppm Mn [29, 30].

Iodine deficiency in Patagonia occurs in people and livestock [4], with goiter rates (1965) among young men reaching 48% [reviewed in 4,9]. Primary deficiency thus may also affect huemul, but is aggravated by Se deficiency causing secondary iodine deficiency. Se deficiency occurs in Patagonia, coinciding with deficient plants and livestock—including severe muscular dystrophy, and soil levels (0.19 ppm) from areas with extant huemul considered very deficient, corroborating deficient huemul [4, 5, 31, 32]. Moreover, of huemul tested in Chile, 73% were Se deficient and 64% severely deficient [9, 32, 33].

#### Marginal habitat causing subnormal antlers and prevalent pathology

Antler phenotypes relate principally to age and nutrition. Development is secondary to optimizing body mass and other needs [34]. Regrowing annually, antlers provide phenomenal tools for interpreting myriad biological and ecological relationships. Huemul with three tines are locally still common (43%, unpubl.), four tines were found repeatedly (> 34 cm), and five tines were also documented previously [34]. Conversely, BONP males show very poor antlers and asymmetries (Additional file 2), indicating nutritional limitations [35–37]. Additionally, high prevalence of unusually severe foot lesions [11] and cases of profound cranial osteopathology occur in BONP [e.g. 28], both indicative of lacking immunocompetence. Lastly, parasite prevalence was highest in BONP (77%) compared to central/northern populations (23% and 12%) [38], also suggesting immunosuppression, and the area produces only a very low density (4.5 deer/km^2^).

#### Parapoxvirus in huemul

The first-ever PPV infection recorded in a single huemul (BONP)—although putative, was concluded as having caused severe foot lesions, also in several other huemul, and thus posing considerable conservation threats in Chile [11]. Significantly, neither bovine papular stomatitis (BPSV) nor pseudocowpox virus (PCPV) usually result in disease as described in those huemul. However, atypical symptoms attributed to PPV in huemul may relate having presumed that PPV was correctly represented by a positive small PCR fragment (192 bp, indicating BPSV/PCPV sequence) obtained with degenerated primers targeting the polymerase gene of PPV. However, this does not prove presence of replicable virus nor its virulence. The choice of degenerate primers to amplify a small PCR fragment is critical for showing presence of the genome of an established virus species. Further research is necessary to exactly define the presence of a PPV species genome and even viable virus in appropriate huemul tissue samples. Moreover, the huemul cases in BONP occurred in areas without cattle, whereas areas in Argentina with coexisting huemul, cattle and red deer have neither resulted in such disease patterns (Additional file 3). Globally, there are very few reported cases of clinical PPV in cervids, considering that ORFV was described early in sheep (1787) and goats (1879). A new variant (PVNZ) was described in New Zealand deer in 1986, which caused including severe disturbance of antler growth [48], but also occurred subclinically in 1% of German deer. For South America, the recent huemul with presumed PPV is first among native and exotic cervids [11, Additional file 3].

#### Nutritional ecology

Recently, the first case of caseous lymphadenitis (CLA) in huemul was considered a potential conservation threat [39]. However, described in 1888, CLA has a well-known global distribution, was common in Argentina by 1913, and obligatory to report in Chile by 1937 [10]. It is not rare in several wild species, and frequently wild fauna form reservoirs. Importantly and also highly relevant, Se deficiency has been shown to negatively affect antibody responses in ruminants against CLA [40].

Reoccurring infections in BONP requires to determine if these have a proximate or ultimate causation. Moreover, the unusual virulence is not a matter of nutritional versus an infectious etiology as proposed by [11], but elicits causes for the unexpected virulence. However, data about diseases and nutritional ecology in huemul [3–6, 8, 9, 32–34, 41] confirms for instance that huemul suffer including from severe Se deficiency in Chile [9, 32, 33]. Moreover, severe osteopathology in one population co-occurs with Se deficiency [5]. Additionally, the present report indicates that Argentine populations with prevalent osteopathology (57% in carcasses, 86% in vivo) [3, 4] are also deficient in key micro-nutrients. Lastly, Se deficiency frequently increases virulence while reducing host immunocompetence, considered a diagnostic bioindicator [23, 40, 47].

#### Importance of habitat quality

Typically, mammals have strong presence in good habitat (source areas), but also occupy marginal areas, and even areas incapable of sustaining populations via reproduction (sink areas). Most remaining huemul subpopulations are either failing to recover, are diminishing, or have recently disappeared, which indicates that their present habitats do not qualify as source areas [9].

Currently huemul occupy mainly forests and shrubby habitats [11], yet this represents only a fraction of historical distributions. Huemul were exterminated in most ecotonal areas, valley bottoms and grasslands as these were settled by men with livestock [41–45]. Extant huemul occur in remote areas like PPS or BONP that were unattractive for humans due to topography, low fertility, winter conditions, and difficult access. Yet, adequate diets determine immunocompetence, both for short-term infections and chronic processes. Commonly, lack of key nutrients causes immunosuppressions [13–18, 20, 21, 46], resulting in marginal or sink areas.

## Conclusions

Argentine populations with prevalent osteopathology are deficient in key micro-nutrients (Se, Cu, Mn). Similarly in BONP, the strong chronic cranial osteopathologies, high disease susceptibility (PPV, parasitism, foot lesions), crippled antlers, and low animal density are indicative of marginal habitat. Primary etiological factors include such nutritional deficiencies and associated immunosuppression.

## Limitations

Some of the presented data is limited by sample size and precludes estimation of the prevalence. Evaluating additional cases regarding osteopathology and micro-nutrients would be important in substantiating the present findings, and should be expanded to other areas and to other populations.

## Supplementary information

**Additional file 1**: Concentration of selenium, copper and manganese in hair from huemul deer from the Protected Park Shoonem, Alto Rio Senguer (province of Chubut, Argentina). Deficiency levels as determined for other cervids, when they are below: < 0.5 ppm Se and < 6.7 ppm Cu [see O’Hara et al. Mineral and heavy metal status as related to a mortality event and poor recruitment in a moose population in Alaska. J Wildl Dis 2001; 37: 509-522], and < 7 ppm Mn [see Franzmann AW, Flynn A, Arneson PD. Alaskan moose hair element values and variability. Comp Biochem Physiol Part A: Physiol 1977;57(3):299-306].

**Additional file 2:** Deficient antler development in huemul. According to antler biology, and in agreement with known species-specific antler phenotypes in huemul, these antler developments from Bernardo O’Higgins National Park (Chile) qualify as subnormal, indicating severe nutritional limitations for the annual cycle of regrowing antlers.

**Additional file 3:** The case of parapoxvirus causing foot disease in huemul. The putative first-ever parapoxvirus (PPV) infection in a single huemul was concluded to have resulted in foot lesions, and would be the first case in exotic or native cervids in South America. The atypical disease symptoms may relate to presuming that PPV was correctly identified. Globally, PPV in cervids occur in most geographical regions, but numbering very few incidences. Instead, the unexpected virulence may relate to nutrition, like severe selenium deficiency in huemul co-occurring with osteopathology. Patagonia has Se deficient plants, livestock, soils, and huemul with osteopathology are deficient in the key micro-nutrients Se, Cu and Mn.

## Data Availability

All data generated or analysed during this study are included in this published article and its supplementary information files.

## Abbreviations

PPS: Protected Park Shoonem
BONP: Bernardo O’Higgins National Park, Chile
PPV: parapoxvirus
ORFV: Orf virus of sheep
PCPV: Pseudocowpox virus
BPSV: Bovine papular stomatitis virus
PVNZ: Pox virus of New Zealand deer
Se: Selenium
Cu: Copper
Mn: Manganese
