# Supplementary material for: Nutrition as an etiological factor causing diseases in endangered huemul deer
Source: BMC Res Notes. 2020 Jun 8;13:276. doi: 10.1186/s13104-020-05122-1 (PMC7282076; doi:10.1186/s13104-020-05122-1)
Supplement: Supplementary file 1 — Additional file 1: Concentration of selenium, copper and manganese in hair from huemul deer from the Protected Park Shoonem, Alto Rio Senguer (province of Chubut, Argentina). Deficiency levels as determined for other cervids, when they are below: < 0.5 ppm Se and < 6.7 ppm Cu [see O’Hara et al. Mineral and heavy metal status as related to a mortality event and poor recruitment in a moose population in Alaska. J Wildl Dis 2001; 37: 509-522], and < 7 ppm Mn [see Franzmann AW, Flynn A, Arneson PD. Alaskan moose hair element values and variability. Comp Biochem Physiol Part A: Physiol 1977;57(3):299-306]. [file 13104_2020_5122_MOESM1_ESM.pdf]

Additional File 1. Concentrations of selenium, copper and manganese in hair from huemul deer from the Protected Park Shoonem, Alto Rio Senguer (province of Chubut, Argentina).

	Concentration in ppm		
	Selenium	Copper	Manganese
female	0.236	4.928	5.294
female	0.333	4.788	1.135
female	0.256	5.57	3.786
male	0.182	6.876	2.179
male	0.275	5.039	1.668
male	0.22	5.452	1.001
female	0.194	4.938	45.03
female	0.336	4.229	66.983
male	0.016	0.266	38.962
male	0.77	7.16	36.079
female	*9.397	5.575	26.887

Deficiency levels as determined for other cervids, when below: <0.5 ppm Se and <6.7 ppm Cu [see O'Hara et al. Mineral and heavy metal status as related to a mortality event and poor recruitment in a moose population in Alaska. J Wildl Dis 2001; 37: 509-522], and <7 ppm Mn [see Franzmann AW, Flynn A, Arneson PD. Alaskan moose hair element values and variability. Comp Biochem Physiol Part A: Physiol 1977;57(3):299-306].

* this value was removed as outlier
