# Supplementary material for: Nutrition as an etiological factor causing diseases in endangered huemul deer
Source: BMC Res Notes. 2020 Jun 8;13:276. doi: 10.1186/s13104-020-05122-1 (PMC7282076; doi:10.1186/s13104-020-05122-1)
Supplement: Supplementary file 2 — Additional file 2: Deficient antler development in huemul. According to antler biology, and in agreement with known species-specific antler phenotypes in huemul, these antler developments from Bernardo O’Higgins National Park (Chile) qualify as subnormal, indicating severe nutritional limitations for the annual cycle of regrowing antlers. [file 13104_2020_5122_MOESM2_ESM.pdf]

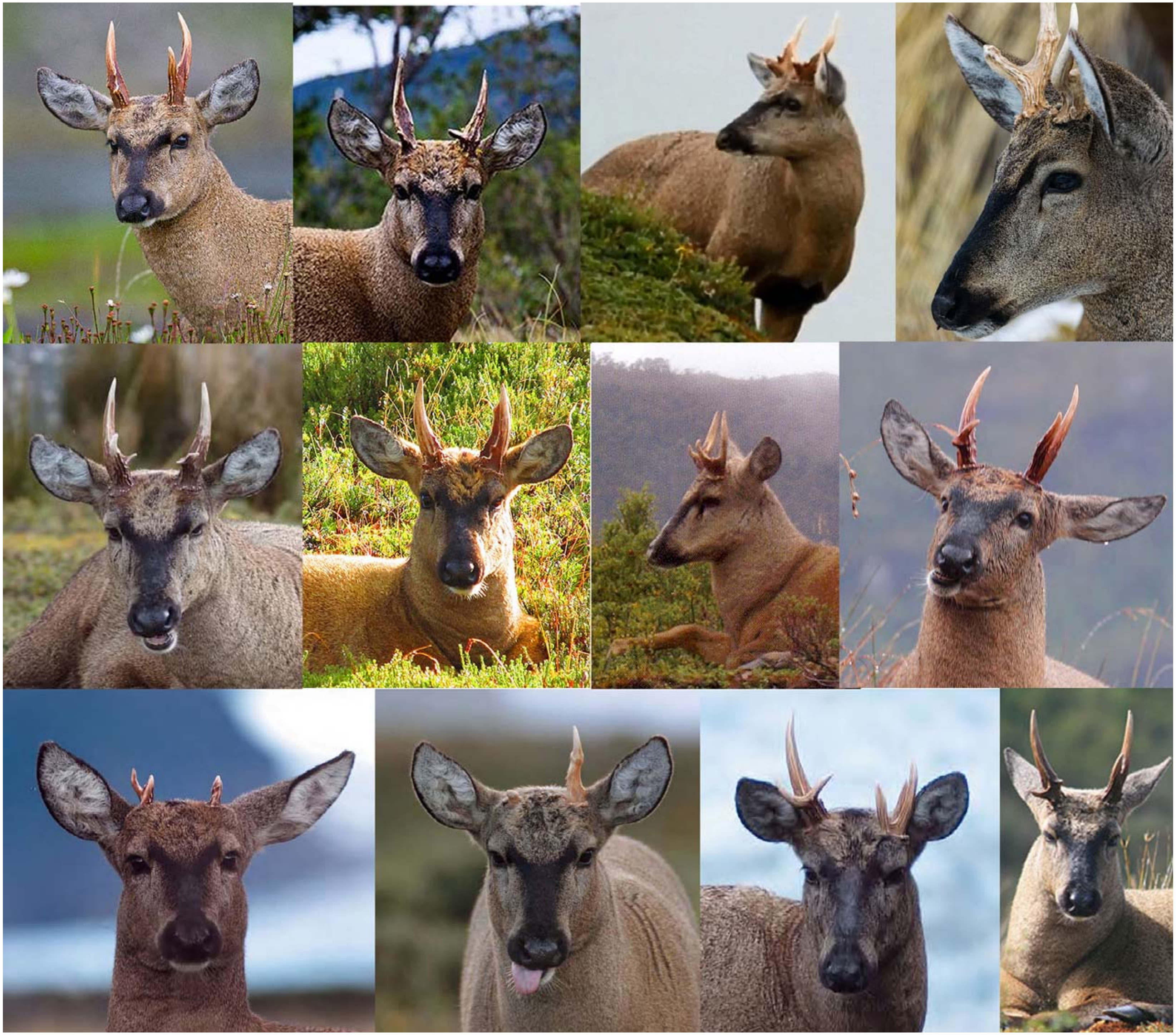

Additional File 1: Deficient antler development in huemul.

Description: According to antler biology, and in agreement with known species-specific antler phenotypes in huemul, these antler developments from Bernardo O'Higgins Natl. Park (Chile) qualify as subnormal, indicating severe nutritional limitations for the annual cycle of regrowing antlers.
