# Supplementary material for: Nutrition as an etiological factor causing diseases in endangered huemul deer
Source: BMC Res Notes. 2020 Jun 8;13:276. doi: 10.1186/s13104-020-05122-1 (PMC7282076; doi:10.1186/s13104-020-05122-1)
Supplement: Supplementary file 3 — Additional file 3: The case of parapoxvirus causing foot disease in huemul. The putative first-ever parapoxvirus (PPV) infection in a single huemul was concluded to have resulted in foot lesions, and would be the first case in exotic or native cervids in South America. The atypical disease symptoms may relate to presuming that PPV was correctly identified. Globally, PPV in cervids occur in most geographical regions, but numbering very few incidences. Instead, the unexpected virulence may relate to nutrition, like severe selenium deficiency in huemul co-occurring with osteopathology. Patagonia has Se deficient plants, livestock, soils, and huemul with osteopathology are deficient in the key micro-nutrients Se, Cu and Mn. [file 13104_2020_5122_MOESM3_ESM.pdf]

The case of parapoxvirus causing foot disease in huemul (*Hippocamelus bisulcus*)

Werner T. Flueck[1,2,3]
[1]Swiss Tropical and Public Health Institute, University Basel, Socinstrasse 57, Basel 4051, Switzerland,
[2]National Council of Scientific and Technological Research (CONICET), Buenos Aires, Argentina,
[3]Argentine National Park Administration, Bariloche
Rolando 699, 8400 Bariloche, Argentina

Introduction

Describing the first-ever parapoxvirus (PPV) infection in a single huemul - although putative, it was concluded that it resulted in foot lesions in this and 23 other individuals from Bernardo O'Higgins National Park, Chile (BONP) [1]. Furthermore, other chronic bone pathology in BONP huemul was discarded [1]. However, the atypical symptomology of PPV described in huemul may relate to having presumed that PPV was correctly represented by a positive small PCR fragment (indicating BPSV/PCPV sequence) obtained with degenerated primers targeting the polymerase gene of PPV. However, this is not evidencing that any pathology was caused by PPV, and less so given the well-recognized ample correlation of small degenerate primers across species and many genotype [2-4].

Appendicular and cranial pathology among huemul

Although PPV infections certainly produce short interactions with hosts, the absence of revisions of legs, nor preservation of appendicular bones and skulls of those diseased huemul in BONP, prevents a comparison with osteopathological processes described in huemul elsewhere [5-10]. Undoubtedly, severe cranial lesions have been documented in Chile [6], including in BONP (Fig. 1). Furthermore, such pathology occurs in subpopulations spread along 1000 km of the Andean mountains [9,10, Frid and Flueck unpubl.]. Lastly, misdiagnoses in live and dead huemul are not uncommon, also because sick huemul frequently pretend a healthy state [9]. For instance, a male reported without health problems was subsequently necropsied and shown to have severe cranial lesions [8].

Parapoxviral disease among huemul

Clinical aspects

The first-ever PPV infection recorded in a single huemul (BONP) was concluded as causing foot lesions in 24 huemul and thus posing considerable conservation threats in Chile [1]. Subclinical infections - amply supported [11-14], must also be considered, and furthermore, a likely polymicrobial etiology cannot be rule out [1]. However, a polyfactorial approach must also include potential roles of nutritional constraints, particularly regarding immunocompetence.

Strikingly, neither bovine papular stomatitis (BPSV) nor pseudocowpox virus (PCPV) usually result in feet nor limbs lesions as described in the BONP huemul, and are considered atypical in livestock and cervids. Moreover, PPV infections rarely become severe, extensive, or fail to spontaneously regress. Additionally, cattle presence and diseased huemul had no spaciotemporal overlap [1], and the general extensive co-occurrences of cattle with huemul (both

in space and time) [15] has not resulted in prior descriptions of such foot lesions. In Argentina, livestock and exotic deer coexist with many remnant huemul, yet few reports exist about BPSV/PCPV in livestock, and none regarding infections among the several hundred thousand of exotic deer.

Cattle with BPSV/PCPV commonly have benign localized inflammations. Asymptomatic livestock with PPV, particularly young animals, are common, and asymptomatic deer (subclinical carriers) had PVNZ [12,16]. Occasionally, severe cases with BPSV or Orf virus of sheep (ORFV) occur, likely linked to immunodeficiency or secondary bacteriosis [12]. Similarly, mortalities from labial ORFV infections are rare [17]. ORFV may occur subclinically in livestock and deer [11-14]: however, lambs and kids can develop severe lesions, with secondary bacteriosis [13]. PPV caused enough oral lesions to affect feeding, and fatal starvation in winter among deer including fawns [17-19]. Nutritional and other factors causing immune-suppression can lead to severe outbreaks even in adult animals as fatal PPV epidemics in reindeer and musk oxen have shown [20-22]. Importantly, selenium deficiency (Se) can affect the evolution of some contemporary RNA viruses and their subsequent viral infectious diseases, and Se-deficient virus-infected hosts supplemented with dietary Se resulted in reduced viral mutation rates and immunocompetence improved [33,34]. They cautioned that environments conducive to chronic dietary Se deprivation should be monitored for bioindicators of incipient viral virulence and subsequent pathogenesis. However, for BONP huemul it remains to be confirmed if PPV is involved, and then determine if either persistent PPV became activated, or if it originated from external sources (fodder, direct or indirect contact to other PPV shedding animals).

Global situation of parapoxvirus infecting cervids

Clinical PPV produces inflammation, stomatitis or typical pox-like lesions in skin or mucous membranes, principally from ORFV/PCPV [12]. Three apparently healthy fawns in Germany (1963) were later found dead due to BPSV/ORFV, which impaired foraging [17]. A weak fawn was found in USA (1983) with head lesions, another fawn with similar lesions in 1984: both had PPV and bacterial infections [23]. In 1986, a new PPV species was detected in New Zealand (PVNZ) [24]. Contagious PCPV was found in Finland during winter 1999-2000 [19]. Hunters handling deer in USA were infected with PCPV [25]. In Alaska, ORFV was first described in 2010 and again in 2012 [26]. Italy had first-ever PVNZ (2011), and oral lesions caused fatal starvation during winter [18]. Analyzing 1764 clinically healthy German deer, 1% contained PVNZ [12]. Additionally, PPV transmission occurred regularly within herds [13]. Overall, PPV in cervids occur in most geographical regions, although numbering very few incidences, considering that ORFV was described early in sheep (1787) and goats (1879). For South America, the recent huemul individual with supposedly PPV is first among native and exotic cervids [1].

Infection of parapoxvirus in huemul

Clinical symptoms among 24 huemul were suggested to stem from PPV supposedly found in one case: morbidity was 40-80% and mortality was 0-40% over 5 years; 50% of cases occurred in autumn; it affected all fawns and juveniles; causing exclusively foot lesions (2-4 feet affected); lesions contained short coccobacilli, Gram positive/negative and spirochete-like bacteria; and it was self-limiting in several individuals [1]. This claimed aggressiveness of PPV in huemul (foot lesions, high morbidity/mortality) are very different from descriptions of PPV in any other hosts,

and demands to elicit if PPV represents a proximate or ultimate cause of host responses. The short gene fragment (192 bp) may indicate presence of PPV, but gives no clue as to the presence of replicable virus, nor its virulence [2-4].

Suggesting that BPSV/PCPV is primarily causing foot lesions in huemul and thus threatening the species in Chile [1] is quite questionable. First, lesions in huemul are totally different (absence of facial/skin lesions, presence of severe foot lesions) than those caused in any other infected host [27]. Moreover, numerous infective agents are commensals, or present only subclinically, as documented for BPSV/PCPV [12,13,16]. Therefore, whereas BPSV/PCPV is considered rather harmless in other cervids worldwide and unknown among exotic deer living in current and prior huemul areas since nearly a century ago, the supposed graveness on huemul in BONP - including death, is contradictory. Lastly, PPV has not been described in any other one of the >100 huemul subpopulations.

Reoccurring PPV infections in huemul from BONP requires to determine its origin. Importantly, the unusual virulence is not a matter of nutritional versus an infectious etiology as proposed by Vila et al. [1], but elicits causes for the unexpected virulence. According to [1], studies on huemul health are scarce and largely anecdotal, and suggested that nutrition as possibly contributing to lesions in huemul in BONP or in Argentina [5] still needs investigations. Yet numerous publications about different huemul health issues were not considered. However, including these (diseases, nutritional ecology) [5-8,15,28-32] confirms, for instance, that huemul suffer including from severe Se deficiency in Chile [28,31,32]. In one population in Chile, 73% were Se deficient and 64% severely deficient. Moreover, the nexus between osteopathology and nutrition for that population exists: co-occurrent Se deficiency and severe cranial osteopathology [6]. Patagonia has Se deficient plants and livestock - including severe muscular dystrophy, and soil levels from areas with extant huemul are considered very deficient. Lastly, the results from hair analyses indicates that Argentine populations with prevalent osteopathology (57% in carcasses, 86% *in vivo*) [5,7] are also deficient in the key micronutrients Se, Cu and Mn.

References

1 Vila AR, Briceño C, McAloose D, Seimon TA, Armién AG, Mauldin EA, et al. Putative parapoxvirus-associated foot disease in the endangered huemul deer (*Hippocamelus bisulcus*) in Bernardo O'Higgins National Park, Chile. PLoS ONE 2019;14(4): e0213667.

2 Elbrecht V, Hebert PD, and Steinke D. 2018. Slippage of degenerate primers can cause variation in amplicon length. Scientific Reports 8:10999

3 Linhart C and Shamir R. 2002. The degenerate primer design problem. BIOINFORMATICS 18(S1):172-180.

4 Buschiazzo E, Beck JS, and Gemmell NJ. 2011. Design and Implementation of Degenerate Microsatellite Primers for the Mammalian Clade. PLoS ONE 6(12):e29582.

5 Flueck WT, Smith-Flueck JM. Age-independent osteopathology in skeletons of a south American cervid, the Patagonian huemul (*Hippocamelus bisulcus*). J Wildl Dis 2008;44(3):636-648.

6 Flueck WT. Osteopathology and selenium deficiency co-occurring in a population of endangered Patagonian huemul (*Hippocamelus bisulcus*). BMC Res Notes 2015;8:330.

7 Flueck WT, Smith-Flueck JM. Troubling disease syndrome in endangered live Patagonian huemul deer (*Hippocamelus bisulcus*) from the Protected Park Shoonem: unusually high prevalence of osteopathology. BMC Res Notes 2017;10:739.

8 Flueck WT. Elusive cranial lesions severely afflicting young endangered Patagonian huemul deer. BMC Res Notes 2018;11:638.

9 Flueck WT, Smith-Flueck JM. The Next Frontier for Recovering Endangered Huemul (*Hippocamelus bisulcus*): How to Avoid Recurrent Misdiagnoses of Health Status and Risks. Animal Prod Sci 2020; in press.

10 Texera WA. Algunos aspectos de la biología del huemul (*Hippocamelus bisulcus*) (Mammalia: Artiodactyla, Cervidae) en cautividad. Ans Inst Pat, Punta Arenas (Chile) 1974;5(1-2):155-188.

11 Chisholm RH, Campbell PT, Wu Y, Tong SYC, McVernon J, Geard N. Implications of asymptomatic carriers for infectious disease transmission and control. Roy Soc Open Sci 2018;5:172341.

12 Friederichs S, Krebs S, Blum H, Lang H, Büttner M. Parapoxvirus (PPV) of red deer reveals subclinical infection and confirms a unique species. J General Virology 2015;96:1446-1462.

13 Tryland M, Klein J, Berger T, Josefsen TD, das Neves CG, Oksanen A, Asbakk K. Experimental parapoxvirus infection (contagious ecthyma) in semi-domesticated reindeer (*Rangifer tarandus tarandus*). Vet Microbiol 2013;162:499-506.

14 Iketani Y, Inoshima Y, Asano A, Murakami K, Shimizu S, and Sentsui H. 2002. Persistent Parapoxvirus Infection in Cattle. Microbiology and immunology 46:285-291.

15 Flueck WT, Smith-Flueck JM. Huemul heresies: beliefs in search of supporting data. 2. Biological and ecological considerations. Ani Prod Sci 2012;52(8):694-706.

16 Roess AA, McCollum AM, Gruszynski K, Zhao H, Davidson W, Lafon N, et al. Surveillance of Parapoxvirus Among Ruminants in Virginia and Connecticut. Zoon Publ Health 2013;60:543-548.

17 Geisel O, Breuer W. Parapox-Infektion bei Rothirschen (*Cervus elaphus*). Zeits Jagdwiss 1993;39:201-206.

18 Scagliarini A, Vaccari F, Turrini F, Bianchi A, Cordioli P, Lavazza A. Parapoxvirus Infections of Red Deer, Italy. Emerg Infect Dis 2011;17(4):684-687.

19 Hautaniemi M, Ueda N, Tuimala J, Mercer AA, Lahdenperä J, McInnes CJ. The genome of pseudocowpoxvirus: comparison of a reindeer isolate and a reference strain. J Gen Virology 2010;91:1560-1576.

20 Büttner, M., C. Von Einem, C. McInnes and A. Oksanen. 1995. Klinik und Diagnostik einer schweren Parapocken-Epidemie beim Rentier in Finnland, Tierärztl. Prax. 23, 614–618.

21 Tryland M, Josefsen TD, Oksanen A, Aschfalk A (2001) Parapoxvirus infection in Norwegian semi-domesticated reindeer (Rangifer tarandus tarandus). Vet Rec. 149(13):394-395.

22 Turid Vikøren, Atle Lillehaug, Johan Åkerstedt, Tord Bretten, ... Morten Tryland. 2008. A severe outbreak of contagious ecthyma (orf) in a free-ranging musk ox (Ovibos moschatus) population in Norway. Vet Microbiol. 2008 Feb 5;127(1-2):10-20.

23 Williams ES, Becerra VM, Thome ET, Graham TJ, Owens MJ, Nunamaker CE. Spontaneous Poxviral Dermatitis and Keratoconjunctivitis in Free-Ranging Mule Deer (*Odocoileus hemionus*) in Wyoming. J Wildl Dis 1985;21(4):430-433.

24 Robinson AJ, Mercer AA. Parapoxvirus of Red Deer: Evidence for Its Inclusion as a New Member in the Genus Parapoxvirus. Virology 1995;208:812-815.

25 Roess AA, Galan A, Kitces E, Li Y, Zhao H, Paddock CD, et al. Novel Deer-Associated Parapoxvirus Infection in Deer Hunters. New Engl J Med 2010;363:2621-2627.

26 Tryland M, Beckmen KB, Burek-Huntington KA, Breines EM, Klein J. Orf virus infection in Alaskan mountain goats, Dall's sheep, muskoxen, caribou and Sitka black-tailed deer. Acta Vet Scand 2018;60(12).

27 Howerth EW, Nemeth KM, Ryser-Degiorgis M. Cervidae. Chpt 6 In: Terio KA, McAloose D, St.Leger J, editors. Pathology of wildlife and zoo animals. London: Academic Press; 2018. p. 149-183.

28 Flueck WT, Smith-Flueck JM. Recent advances in the nutritional ecology of the Patagonian huemul: implications for recovery. Animal Prod Sci 2011;51(4):311-326.

29 Flueck WT, Smith-Flueck JM, Mionczynski J, Mincher BJ. The implications of selenium deficiency for wild herbivore conservation, a review. Europ J Wildl Res 2012; 58:761-780.

30 Flueck WT, Smith-Flueck JM. Huemul heresies: beliefs in search of supporting data. 1. Historical and zooarcheological considerations. Ani Prod Sci 2012;52(8):685-693.

31 Flueck WT, Smith-Flueck JM, Mincher BJ, Winkel LHE. An Alternative Interpretation of Plasma Selenium Data from Endangered Patagonian Huemul Deer (*Hippocamelus bisulcus*). J Wildl Dis 2014;50:1003-1004.

32 Flueck WT, Smith-Flueck JM, Mincher BJ, Winkel LHE. Soil selenium levels corroborate direct evidence of selenium deficiency in endangered Patagonian huemul deer (*Hippocamelus bisulcus*). In: Ma J, Zhang M, Halbrook R, Liu B, Zhang W, editors. Proceedings of the 8th International Deer Biology Congress. Harbin, China: Northeast Forestry University; 2014. pp. 52-53.

33 Harthill M. Review: Micronutrient selenium deficiency influences evolution of some viral infectious diseases. Biol Trace Element Res 2011;38(13):77-83.

34 Beck MA, Levander OA, Handy J. Selenium deficiency and viral infection. J Nutr 2003;133:1463S–1467S.

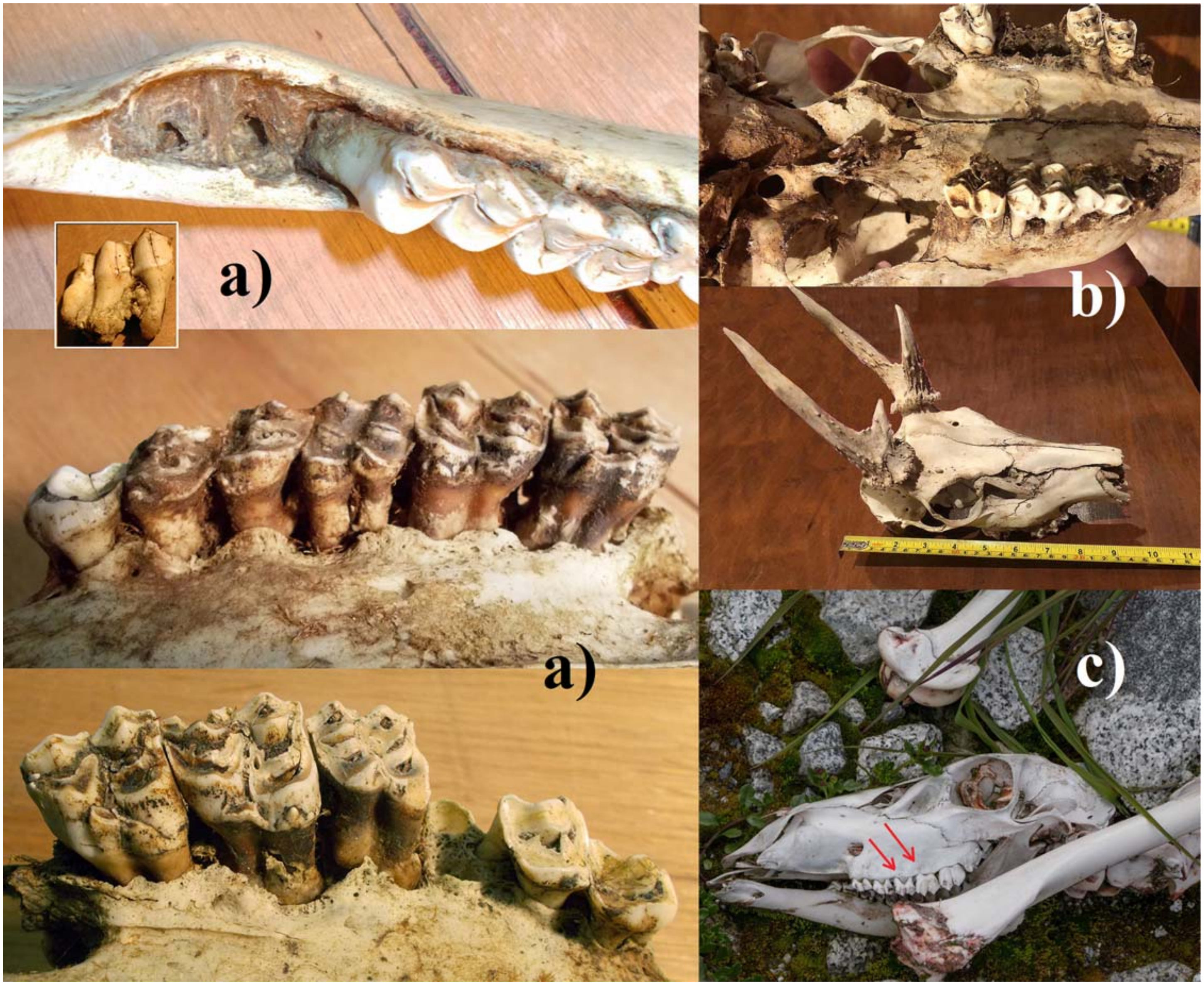

Figure 1
Huemul heads with pathological lesions from Bernardo O'Higgins National Park (Chile): a) head of female observed in 1993: with perforations on buccal and palatal sides of maxillary bone, exposing root apices of premolar and molar teeth; exposed roots of maxillary teeth from generalized erosion and resorption of bone resulting in enlarged dental alveoli; crystalline deposits on tooth roots (insert); resorption as well as osteomyelitic thickening of mandibular body. b) Male head from 2017: perforations on buccal and palatal sides of maxillary bone, exposing root apices of premolar and molar teeth; exposed roots of maxillary teeth from generalized erosion and resorption of bone resulting in enlarged dental alveoli (above). Antler development (below) qualifies as subnormal, indicating severe nutritional limitations for the annual cycle of antler regrowth. c) Juvenile female from 2019: resorption on buccal side of maxillary bone, exposing roots of premolar and molar teeth (Ultima Patagonia 2019, www.centre-terre.fr Accessed 10 July 2019).
